# Dissecting the Genotypic Variation of Growth Responses to Far-Red Radiation in Tomato

**DOI:** 10.3389/fpls.2020.614714

**Published:** 2021-01-13

**Authors:** Yongran Ji, Theoharis Ouzounis, Henk J. Schouten, Richard G. F. Visser, Leo F. M. Marcelis, Ep Heuvelink

**Affiliations:** ^1^Horticulture and Product Physiology, Department of Plant Sciences, Wageningen University & Research, Wageningen, Netherlands; ^2^Plant Breeding, Department of Plant Sciences, Wageningen University & Research, Wageningen, Netherlands

**Keywords:** far red, genotypic variation, growth analysis, LED lighting, *Solanum lycopersicum*

## Abstract

The recent development of light-emitting diodes (LEDs) and their application in modern horticulture stimulated studies demonstrating that additional far-red (FR) radiation (700–800 nm) increases plant dry mass. This effect of FR has been explained by improved photosynthesis and/or plant architecture. However, the genotypic variation in this response is largely unknown. Here, we aim to explore and explain the genotypic variation in growth responses to additional FR. We expected the genotypic variation in the responses of plant dry mass to additional FR. Further, we hypothesized that a significant improvement of both net assimilation rate (NAR) and leaf area ratio (LAR) is responsible for a strong dry mass increase under additional FR, while some genotypes respond only marginally or even negatively in NAR or LAR under FR, thus resulting in a weak FR effect on plant dry mass. To test these hypotheses, we grew 33 different tomato genotypes for 21 days with 0, 25, or 100 μmol m^–2^ s^–1^ of FR added to a common white + red LED background lighting of 150 μmol m^–2^ s^–1^. Genotypes responded similarly with respect to plant height, stem dry mass, and shoot:root ratio; i.e., they all increased with increasing FR. However, the response of total plant dry mass varied among genotypes. We categorized the genotypes into three groups (strongly, moderately, and weakly responding groups) based on their relative response in total plant dry mass to FR. Growth component analysis revealed that the strongly responding genotypes increased strongly in NAR rather than LAR. The weakly responding genotypes, however, showed a substantial increase in LAR but not NAR. The increase in LAR was due to the increase in specific leaf area. Leaf mass fraction, which is the other component of LAR, decreased with FR and did not differ between groups. In conclusion, tomato genotypes that increased strongly in NAR in response to FR were able to achieve a more substantial increase in dry mass than did other genotypes. This is the first study to explain the differences in growth responses of a large number of tomato genotypes toward FR in their light environment.

## Introduction

Far-red (FR) radiation (700–800 nm) is an important light signal perceived by plants via the phytochrome photoreceptor family. Phytochromes exist as two photo-interconvertible isoforms, that is, the red (R)-absorbing biologically inactive P_r_ and the FR-absorbing active P_fr_ (Chen et al., 2005). A low R:FR ratio causes the equilibrium between the two isoforms of phytochromes to shift toward Pr, resulting in a set of morphological and physiological changes collectively known as the shade-avoidance syndrome (SAS). SAS responses such as stem elongation, leaf hyponasty, and flowering acceleration enable the plant to compete for more light capture and to secure reproductive success, as decreased R:FR ratio occurs naturally when plants are shaded (Huber and Wiggerman, 1997; Devlin, 1998; Yang et al., 2016; Michaud et al., 2017).

In the past decades, light-emitting diodes (LEDs) gained popularity in modern horticulture, a development that stimulated the study of spectral effects on plant growth and development. Plant photosynthesis is driven by photosynthetically active radiation (PAR; 400–700 nm). FR is not commonly considered to be part of PAR, as monochromatic FR drives neither CO_2_ assimilation nor O_2_ evolution from photosynthesis (Kono et al., 2020). When added to PAR, however, FR radiation may increase not only yield (Ji et al., 2019, 2020) but also total plant biomass production (Li and Kubota, 2009; Park and Runkle, 2017; Zhen and van Iersel, 2017). Much effort has been made to explain FR-enhanced plant growth. It has been found that FR-induced changes in plant architecture increase light interception (Kalaitzoglou et al., 2019). For a long time, FR effect on leaf photosynthesis has been described as the Emerson enhancement effect: radiation at shorter wavelengths enhances the quantum yield of radiation at longer wavelengths (Emerson et al., 1957; Emerson and Rabinowitch, 1960; Govindjee et al., 1964). Several recent studies revisited this concept and proposed the reverse interpretation: FR radiation enhances the quantum yield of PAR (Zhen and van Iersel, 2017). Furthermore, Zhen and Bugbee (2020) demonstrated in an experiment with 14 species of both C_3_ and C_4_ crops that FR can be as efficient in driving photosynthesis as PAR, not by itself but when provided in addition to PAR.

Modern horticultural production can benefit from a deeper understanding of plants’ responses to different light spectra. More importantly, it is crucial to explore the genotypic variation in such responses. For example, Ouzounis et al. (2016) showed genotypic differences in growth and physiological parameters when plants were grown in a red LED background with or without 12% of blue LED lighting. Plant’s response to FR is a new way to increase crop production and resource use efficiency (Demotes-Mainard et al., 2016). However, the genotypic variation in plants’ responses to additional FR is largely unknown due to the often-limited numbers of genotypes used in FR-related research. Here, we aim to evaluate and explain the similarities and differences between tomato genotypes in growth responses under additional FR. We hypothesize that not all genotypes respond the same way in their dry mass production under additional FR. Further, we hypothesize that this variation is the result of different morphological or physiological responses in the components of dry mass production under additional FR. To test these hypotheses, we conducted a climate chamber experiment where 33 tomato genotypes were grown for 21 days with 0, 25, or 100 μmol m^–2^ s^–1^ of FR added to a common white + red LED lighting background of 150 μmol m^–2^ s^–1^. Growth component analysis, which subdivides growth into underlying morphological and physiological components (Jolliffe and Courtney, 1984), is a useful tool to dissect the effect of FR on dry mass production (Higashide and Heuvelink, 2019). Here, growth components such as relative growth rate (RGR), net assimilation rate (NAR), leaf area ratio (LAR), specific leaf area (SLA), and leaf mass fraction (LMF) were determined, and the contribution of the different growth components to the genotypic variation in growth response was evaluated.

## Materials and Methods

### Plant Materials and Growth Conditions

The experiment was conducted in a fully controlled climate chamber at Wageningen University (Wageningen, Netherlands). The air temperature was maintained at 22°C, and the relative humidity was 70%. In this climate chamber, seeds of 33 tomato (*Solanum lycopersicum*, Table 1) genotypes, varying in genetic background and morphological traits (Aflitos et al., 2014), were germinated under white fluorescent light (Philips, Eindhoven, Netherlands) with 16 h photoperiod. Ten days after sowing, eight uniform seedlings of each genotype were individually transplanted into 10.5-cm-diameter plastic pots filled with sterilized river sand and placed onto the experimental bench equipped with an ebb-and-flow system. The plants were irrigated daily with nutrient solution (electrical conductivity 2.0 dS m^–1^, pH 5.5) containing 1.2 mM of NH_4_^+^, 7.2 mM of K^+^, 4.0 mM of Ca^2+^, 1.8 mM of Mg^2+^, 12.4 mM of NO_3_^–^, 3.3 mM of SO_4_^2–^, 1.0 mM of PO_4_^2–^, 35 μM of Fe^3+^, 8.0 μM of Mn^2+^, 5.0 μM of Zn^2+^, 20 μM of B, 0.5 μM of Cu^2+^, and 0.5 μM of MoO_4_^2–^.

**TABLE 1:** List of genotypes used in the experiment and their relative response in total dry mass to increasing far red and their corresponding growth response groups.

**No.**	**Code**	**Name**	**Source or identification^1^**	**Relative response (μmol^–1^ m^–2^ s^–1^)**	**Group**
1	RF-1	Moneymaker	LA2706/EA00840/EA02936/	0.0073	Strong
2	RF-102		LA4133/TR00026	0.0092	Strong
3	RF-15	Momotaro	TR00003	0.0075	Strong
4	RF-16	Rote Beere	LYC11/EA01965/CGN15464	0.0177	Strong
5	RF-2	Alisa Craig	LA2838A/EA01101/EA00240/	0.0094	Strong
6	RF-23		PI272654/EA05170	0.0104	Strong
7	RF-29	Black Cherry	LA4451/EA00027	0.0080	Strong
8	RF-3	Gardeners delight	EA06086/PI406760	0.0109	Strong
9	RF-7	Katinka Cherry	EA00375	0.0083	Strong
10	RF-94	Marmande	TR00022	0.0089	Strong
11	RZ-CAP	Cappricia	Rijk Zwaan	0.0093	Strong
12	BJ-HB1	Hybrid-1	Bejo Zaden	0.0073	Moderate
13	RF-11	Allround	LA2463/LYC1365/EA02617	0.0050	Moderate
14	RF-20		LYC3153/EA03221	0.0055	Moderate
15	RF-22		PI129097/EA04710	0.0050	Moderate
16	RF-226		EA05721	0.0070	Moderate
17	RF-27	Cal J Tm VF	EA02054/CGN20815	0.0039	Moderate
18	RF-34	Tiffen Mennonite	EA01088	0.0038	Moderate
19	RF-40	ES 58 Heinz	LYC1410/EA02655	0.0063	Moderate
20	RF-43		LYC2910/EA03058/T115	0.0071	Moderate
21	RF-89	Brandywine	EA01019	0.0053	Moderate
22	RF-97	Watermelon beefsteak	EA01640	0.0073	Moderate
23	BJ-HB2	Hybrid-2	Bejo Zaden	-0.0014	Weak
24	N-9008	Foundation	Nunhems	0.0037	Weak
25	N-9098	9098	Nunhems	0.0004	Weak
26	N-FM001	FM001	Nunhems	0.0009	Weak
27	RF-103		LA1421/TR00027	-0.0021	Weak
28	RF-206		EA00915	0.0034	Weak
29	RF-229		EA05979	0.0026	Weak
30	RF-4	Rutgers	LA1090/EA00465	0.0003	Weak
31	RF-91	Giant Belgium	EA01037	-0.0006	Weak
32	RF-93	Kentucky Beefsteak	TR00021	0.0037	Weak
33	RZ-CAL	Caldino	Rijk Zwaan	0.0036	Weak

*^1^Identification starting with “EA,” “LA,” “LYC,” “PI,” and “TR” are genotypes registered by “EU-SOL tomato core collection database” (Aflitos et al., 2014), while others are provided by the corresponding company.*

### Light Treatment

A deep red + white light at 150 μmol m^–2^ s^–1^ with 0.16 W m^–2^ of UV-B of was used as the control light treatment, and two light treatments were applied from transplanting (10 days after sowing). There were three FR treatments: 0, 25, or 100 μmol m^–2^ s^–1^ of FR radiation was added to a common background of red + white LED light of 150 μmol m^–2^ s^–1^ with 0.16 W m^–2^ of UV-B. The UV-B radiation was included to mimic the UV dosage in natural solar radiation. All lighting was provided by LED modules (Control: 3× GreenPower LED-TL-DR/W-MB-VISN; FR: 15 or 60× GreenPower LED-RM-FR, Philips, Eindhoven, Netherlands) except for UV-B (2× TL 20W/12 RS Ultraviolet-B, Philips). Light modules were placed 1.3 m above the experimental bench. Spectral distribution (Supplementary Figure S1) and photon flux density (PFD) of the LED lighting (Table 2) was measured at canopy height at transplanting with a spectroradiometer (USB 2000+ UV-VIS, Ocean Optics, Duiven, Netherlands) on 30 evenly distributed spots on the experimental bench. Based on these measurements, values of phytochrome photostationary state (PSS) were calculated as described in Sager et al. (1988).

**TABLE 2:** Photosynthetic photon flux density (PPFD), photon flux density (PFD) of far red, red:far red ratio, and phytochrome photostationary state (PSS) of the LED light measured at the top of canopy.

**Light treatment**	**PPFD (μmol m^–2^ s^–1^)**	**Far red (μmol m^–2^ s^–1^)**	**R:FR^1^**	**PSS**
White + red	151 ± 2^2^	3 ± 0.2	35 ± 1.3	0.87
White + red + 25 FR	152 ± 3	28 ± 0.9	3 ± 0.1	0.80
White + red + 100 FR	155 ± 5	95 ± 3.6	1 ± 0.1	0.69

*^1^For the calculation of ratios, PFD was integrated over 100-nm intervals for red (600–700 nm) and far red (700–800 nm). ^2^All values are means ± standard error of means (SEM). SEM of PSS was very small (<0.001) and therefore not shown.*

### Data Collection

#### Non-destructive Measurement

After 14 days of growth, stomatal conductance and chlorophyll index on the first fully expanded leaf of each experimental plant were determined. Stomatal conductance was measured with a SC-1 leaf porometer (Decagon Devices, Inc., Pullman, WA, United States), and chlorophyll index was measured using a Dualex leaf-clip sensor (Force-A, Orsay, France). For the chlorophyll measurement, the values measured from both sides of the leaf were averaged.

#### Destructive Measurement

After 21 days from transplanting, a final destructive harvest was carried out. Each experimental plant was carefully cleaned to remove any remaining river sand from the roots. Excess water was wiped clean with tissue paper, and the plant height was measured immediately, after which the plant was separated into roots, stem, and leaves. Total leaf area was measured using an area meter (LI-3100, Li-Cor Biosciences, Lincoln, NE, United States). Leaves, stems, and roots were dried in a ventilated oven for 72 h at 105°C to obtain the dry mass. For each genotype, the initial dry mass at transplanting was measured using seedlings of each genotype germinated in the same conditions as the experimental plants.

### Growth Component Analysis

**FIGURE 1:**
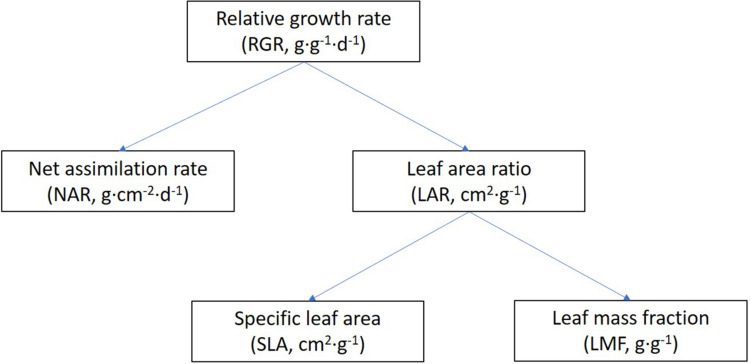
General scheme of a growth component analysis of relative growth rate. Abbreviations and units are included in brackets. RGR is the product of NAR and LAR, and LAR is the product of SLA and LMF.

A linear relation was fitted between the total dry mass and PFD of FR for each genotype. Then, the relative response of each genotype was calculated as the ratio between the slope of this line and the absolute total plant dry mass (TDM) in the control light treatment. All 33 genotypes were then ranked by their relative response to increasing FR in total dry mass, and three response groups were distinguished, i.e., the strongly, moderately, and weakly responding groups, with 11 genotypes in each group. Effects of additional FR on RGR were analyzed using a growth component analysis, which separates RGR from its underlying components (Figure 1) (Hunt et al., 2002). RGR is the product of NAR and LAR, as shown in Eq. 1. NAR was calculated by dividing RGR by LAR.

(1) N⁢A⁢R=R⁢G⁢R/L⁢A⁢R

Relative growth rate was calculated according to Eq. 2 using the initial plant dry mass (DW_initial_) and the final plant dry mass (DW_final_) of each plant after 21 days of growth.

(2) RGR=(ln(DW)final-ln(DW)initial)/21

Further, LAR was analyzed as the product of SLA and LMF as indicated by Eq. 3.

(3) L⁢A⁢R=S⁢L⁢A*L⁢M⁢F

Leaf area ratio, SLA, and LMF were calculated from the measured total leaf area (LA_plant_), final plant dry mass (DW_final_), and leaf dry mass per plant (DW_leaf_) using Eqs 4–6.

(4) LAR=LA/plantDWfinal

(5) SLA=LA/plantDWflea

(6) LMF=DW/leafDWfinal

### Experimental Setup and Statistical Analysis

Each experiment with one light treatment was conducted consecutively in the same fully controlled climate room. For each light treatment, eight blocks were designed according to the light distribution over the bench, and one plant per genotype was randomly placed in each block. The experiment with 25 μmol m^–2^ s^–1^ of FR was repeated in time for one extra time (again with eight blocks). To prevent border effects, *S. lycopersicum* cv. Moneymaker plants were grown around the experimental plants as border plants. Responsiveness of plant dry mass and RGR to additional FR was quantified as the slope of a linear regression with the FR PFD as the regressor. For the growth component analysis, statistical differences for the FR effect in each group were tested with paired sample *t*-test (genotypes defining the pairs). All statistics were performed in Genstat (18th Edition, VSN International Ltd., Hemel Hempstead, United Kingdom) at α = 0.05.

## Results

### Effect of Far-Red Radiation on Growth Parameters

**FIGURE 2:**
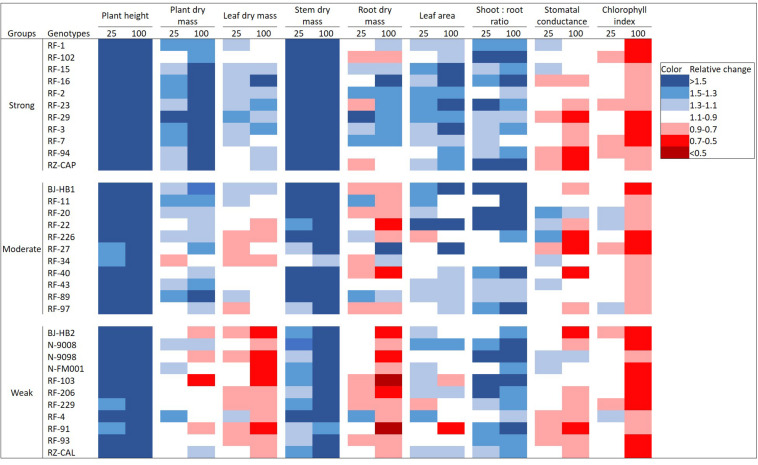
Effects of adding 25 or 100 μmol m^–2^ s^–1^ of far-red (FR) radiation on plant height, plant dry mass, leaf dry mass, stem dry mass, root dry mass, leaf area, shoot:root ratio, stomatal conductance, and chlorophyll index in 33 tomato genotypes. Genotypes were categorized into three groups (strongly, moderately, and weakly responding groups) based on their relative responses in total plant dry mass to FR. Color scales represent relative changes of parameters when compared with the control light treatment without FR, with blue indicating an increase under FR and red representing a decrease.

Effects of additional FR varied among genotypes and among growth parameters studied (Figure 2). Plant height, stem dry mass, and shoot:root ratio increased in all genotypes with increasing FR. Chlorophyll index showed a minor decrease by adding 25 μmol m^–2^ s^–1^ of FR and a stronger and universal decrease in all genotypes by adding 100 μmol m^–2^ s^–1^ of FR. Responses of plant dry mass, leaf dry mass, root dry mass, and leaf area to increasing FR varied among genotypes. For plant dry mass, 58% of the genotypes showed a positive response under 25 μmol m^–2^ s^–1^ of FR, and this percentage increased to 70% under 100 μmol m^–2^ s^–1^ of FR. For leaf dry mass and root dry mass, about 30–40% of the genotypes responded positively to increasing FR, most of which belong to the strongly responding group (genotypes whose total dry mass increased relatively strong with FR). For stomatal conductance, half of the genotypes responded positively to 25 μmol m^–2^ s^–1^ of additional of FR, while this fraction decreased to 21% under 100 μmol m^–2^ s^–1^ of additional FR. Absolute numbers of each parameter are shown in Supplementary Table S1.

### Growth Component Analysis

**FIGURE 3:**
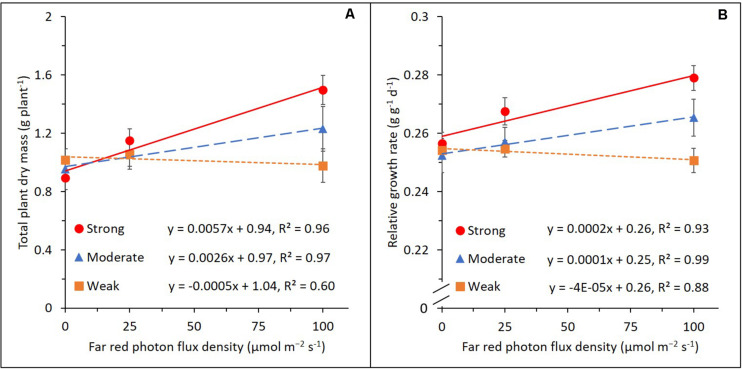
Effects of adding 25 or 100 μmol m^–2^ s^–1^ of far-red (FR) radiation on total plant dry mass **(A)** and relative plant growth rate **(B)** in the strongly (red circle), moderately (blue triangle), and weakly (orange rectangle) responding groups of genotypes. Lines represent linear regression. Error bars represent standard error of means (*n* = 8 for 0 and 100 μmol m^–2^ s^–1^ of FR and *n* = 16 for 25 μmol m^–2^ s^–1^ of FR).

In order to explain the variation in the FR effect on plant dry mass production, we categorized the genotypes into three groups (i.e., strongly, moderately, and weakly responding groups; 11 genotypes in each group) based on their relative response to increasing FR in TDM (Figure 3A and Table 1). RGR, which is a common parameter used for growth component analysis, showed a similar pattern to TDM in response to increasing FR (Figure 3B). Slopes of the regression models fitted for both total dry mass and RGR showed significant differences between the three groups.

**FIGURE 4:**
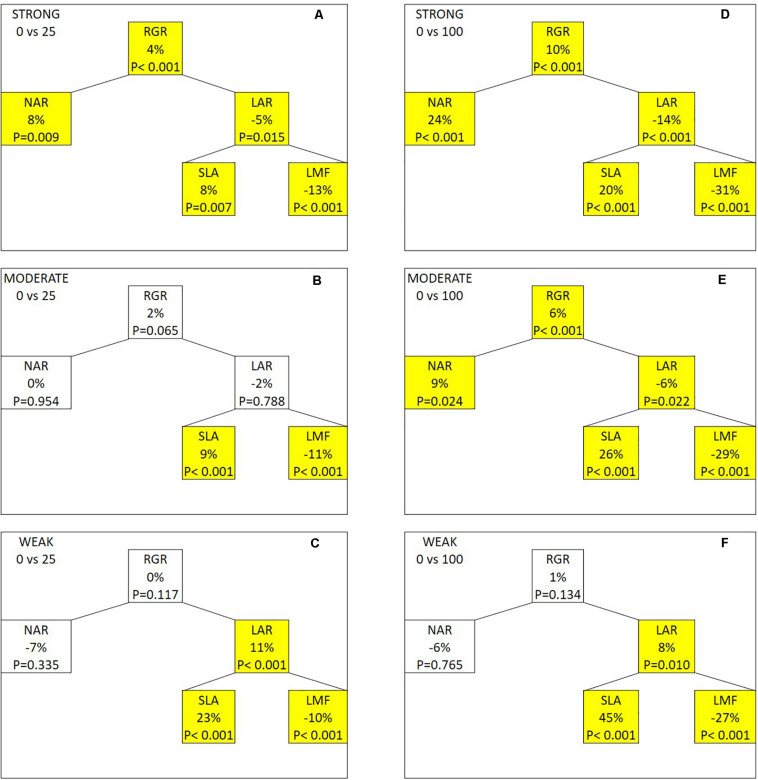
Effects of adding 25 **(A–C)** or 100 μmol m^–2^ s^–1^ of far-red (FR) radiation **(D–F)** on the growth components in the strongly, moderately, and weakly responding groups of genotypes. Abbreviations in this figure: RGR, relative growth rate; NAR, net assimilation rate; LAR, leaf area ratio; SLA, specific leaf area; LMF, leaf mass fraction. The percentage represents the relative change in the components when compared between the FR treatment and the control treatment. *P*-value of the paired *t*-test is indicated in each component with a significant difference (*P* < 0.05) being highlighted in yellow.

This similarity facilitates using a growth component analysis of RGR to explain the genotypic variation in the FR effect on total dry mass (Figure 4). When 25 μmol m^–2^ s^–1^ of FR was provided, RGR and NAR increased in the strongly responding group, while both were not significantly affected in the moderately and weakly responding groups. LAR showed an opposite response to FR with a decrease in the strongly responding group and an increase in the weakly responding group. LAR was further divided into SLA and LMF. LMF decreased in all three groups by a comparable magnitude, while SLA increased with FR with the weakly responding group showing the strongest increase, followed by moderately and strongly responding groups. Similar responses of the growth components were observed when additional FR increased from 25 to 100 μmol m^–2^ s^–1^. Here, an additional 100 μmol m^–2^ s^–1^ of FR resulted in a significant increase in RGR and NAR in the strong and moderate groups, while those in the weak group were not statistically significant. Also, 100 μmol m^–2^ s^–1^ of FR decreased the LAR in the strong and moderate groups while increasing that in the weak group. This was due to the difference in the increasingly large response in SLA from strong to weak group. LMF strongly decreased with FR with only marginal differences between the three groups. For all parameters, there was a clear dosage effect as the responses became more substantial as FR increased from 25 to 100 μmol m^–2^ s^–1^. The absolute numbers of the parameters used in the component analysis are presented in Supplementary Table S2.

## Discussion

### Genotypic Similarities and Variations in Growth Response to Far Red

This study is the first to analyze the differences in growth responses of a large number of tomato genotypes toward FR in their light environment (Figure 2). The most distinct response to FR in many species is stem elongation, which has been reported in many species (Kasperbauer, 1971; Franklin and Quail, 2010; Kalaitzoglou et al., 2019; Shibuya et al., 2019). In agreement with this, we observed a universal increase of plant height in all 33 genotypes, and this increase in plant height was dosage dependent. Corresponding to the FR-induced stem elongation, stem dry mass also increased with FR in all genotypes, and this agreed well with other studies (Ji et al., 2019; Zhang et al., 2019). In general, responses of leaf growth to FR may vary between species and genotypes (Casal and Smith, 1989). Also in tomato, both positive (Cao et al., 2018; Zhang et al., 2019) and negative (Ji et al., 2019; Kim et al., 2019) effects of FR on leaf dry mass have been reported. Similarly, we observed that the response of leaf dry mass to FR varied among genotypes, ranging from negative to positive response when grown with FR, with a negative response being more frequent. FR stimulates the dry mass to be distributed more to the above ground, thus increasing the shoot:root ratio (Kasperbauer, 1987; Lee et al., 2016; Cao et al., 2018). In line with these results, we observed that all genotypes responded positively to increasing FR in shoot:root ratio, which may be a combined result of higher shoot (mainly stem) dry mass and a lower root dry mass. In this study, we noticed that the increase in shoot:root ratio for the strongly responding genotypes was likely due to an increase in shoot dry mass that was stronger than the increase in root dry mass. For moderately and weakly responding genotypes, this was a result of an increase in shoot dry mass combined with a decrease in root dry mass. Interestingly, FR decreased the chlorophyll index, which indicates that FR reduces chlorophyll content and suggests that photosynthetic capacity may be reduced. Similarly, decrease in chlorophyll content was also reported both in young tomato and fruiting tomato plants (Cao et al., 2018; Kalaitzoglou et al., 2019; Kim et al., 2019) as well as other crops (Tucker, 1981; Casal and Aphalo, 1989; Li and Kubota, 2009). Furthermore, despite a trend of increased TDM due to FR, the genotypic variation in the response was very noticeable when comparing the magnitude of this FR effect.

### Genotypes Achieved a Stronger Increase in Dry Mass Production by the Increase in Net Assimilation Rate

We categorized the genotypes into three groups (i.e., strongly, moderately, and weakly responding groups) based on their relative response in TDM to FR (Table 1) to conduct a growth component analysis based on the breakdown of RGR (Hunt et al., 2002). RGR is the product of NAR and LAR. The strongly responding genotypes substantially increased their RGR under additional FR, followed by the moderately responding genotypes, while the weakly responding genotypes showed no significant changes in their RGR under FR (Figure 4). The increase in RGR of the strongly responding genotypes under FR was the result of an increase in NAR, but not in LAR, as it decreased with FR. FR was reported by Ji et al. (2019) and Kalaitzoglou et al. (2019) to increased SLA. Here, we found that the weakly responding genotypes showed a stronger increase in SLA than did other genotypes. LMF, the other component of LAR, was significantly decreased for all groups, and the response did not differ between groups and was only dependent on the amount of FR. The dry mass partitioning between organs is regulated by the relative sink strength of the organs (Marcelis, 1996). The decreased LMF may be due to the strong enhancement of stem sink strength under FR, causing less dry mass to be partitioned to the leaves. For both the strongly and weakly responding groups, their responses to FR were in accordance with the known SAS responses. Our result suggests that when grown under additional FR, tomato plants are not likely to be able to increase NAR and LAR simultaneously, and that the genotypes with a strong increase in NAR under FR allowed them to achieve a stronger increase in RGR than did other genotypes.

### Possible Mechanism of Far-Red Enhancement in Net Assimilation Rate

One explanation for the FR-increased NAR may be that the morphology of plants grown with FR contributed to better vertical distribution of light. FR increases the internode length in tomato, which may lead to a more open plant architecture. Indeed, up to 10% of increase in canopy photosynthesis was achieved in a model simulation by increasing internode length in tomato (Sarlikioti et al., 2011). Also, NAR represents largely the net carbon gain from photosynthesis (Poorter and Remkes, 1990). FR enhances the quantum yield of PAR (400–700 nm) in various species (Zhen and van Iersel, 2017; Zhen and Bugbee, 2020). Such an improvement in photosynthesis agrees with our finding that FR increases NAR. However, their studies focused on short-term light treatments. Experiments with plants grown or adapted to additional FR showed varying results. For example, Kalaitzoglou et al. (2019) found that a 4-week growth period with additional FR resulted in a higher net leaf photosynthesis rate (*A*) when 50 μmol m^–2^ s^–1^ of FR was added to 150 μmol m^–2^ s^–1^ PAR. Cao et al. (2018), however, reported no significant differences in *A* using a comparable spectrum. In addition, no significant FR effect on *A* was reported for tomato plants grown with prolonged exposure to additional FR until fruiting stage (Ji et al., 2019; Zhang et al., 2019). This may indicate that the short-term FR enhancement in photosynthesis cannot fully explain the increase in NAR either, especially when considering the decrease in chlorophyll index (Figure 2; Li and Kubota, 2009; Cao et al., 2018; Kalaitzoglou et al., 2019) and a decreased photosynthetic capacity (Ji et al., 2019). FR may also reduce the photosynthetic rate via the *phyB*-mediated downregulation of genes such as *FAMA* and *TMM* in *Arabidopsis*, leading to the reduction of stomata development (Boccalandro et al., 2009). This reduction, however, may be compensated by the increase in water-use efficiency. To date, there is still insufficient evidence to fully dissect the effect of FR on the NAR due to the complex interaction between the underlying morphological and physiological components. We do, however, speculate that the effect of FR (positive, neutral, or negative) on net photosynthesis rate, light interception, and light distribution varies and that it is the combined effect that determines the NAR.

## Conclusion

Genotypes responded similarly with respect to plant height, stem dry mass, and shoot:root ratio. However, the response of TDM varied among genotypes. Here, we demonstrated that it was the differences in genotype’s responses in NAR and LAR that explain the genotypic variation in response to total dry mass. Genotypes with a strong increase in RGR with increasing FR showed a strong increase in NAR rather than LAR. The weakly responding genotypes, however, showed a substantial increase in LAR but not NAR. The genotypic differences in the increase in LAR were mainly due to the genotypic differences in the increase in SLA, while the responses of LMF to FR were conserved between genotypes.

## Data Availability Statement

The raw data supporting the conclusions of this article will be made available by the authors, without undue reservation.

## Author Contributions

YJ wrote the manuscript. YJ and TO conducted the experiment and conducted the analysis. LM and EH initiated this work and obtained funding for this research, provided guidance in the experimental design and data analysis, and provided critical comments on the manuscript. HS and RV provided critical comments to the overall structure of the manuscript. All authors reviewed and approved the final manuscript.

## Conflict of Interest

The authors declare that the research was conducted in the absence of any commercial or financial relationships that could be construed as a potential conflict of interest.
