# Supplementary material for: Dissecting the Genotypic Variation of Growth Responses to Far-Red Radiation in Tomato
Source: Front Plant Sci. 2021 Jan 13;11:614714. doi: 10.3389/fpls.2020.614714 (PMC7838372; doi:10.3389/fpls.2020.614714)
Supplement: Supplementary file 1 [file Data_Sheet_1.docx]

# Supplementary Material

Table S1 Plant height, plant dry mass, leaf dry mass, stem dry mass, root dry mass, leaf area, shoot: root ratio, stomatal conductance and chlorophyll index of all genotypes grown with 0, 25 or 100 μmol m^−2^ s^−1^ of far red measured after 21 days of growth.

| Group | Genotype | Plant height | | | Leaf area | | | Stomatal conductance | | | Chlorophyll index | | |
| --- | --- | --- | --- | --- | --- | --- | --- | --- | --- | --- | --- | --- | --- |
|  |  | (cm) | | | (cm2) | | | (mmol m-²s-1) | | | (µg cm²) | | |
|  | Far red | 0 | 25 | 100 | 0 | 25 | 100 | 0 | 25 | 100 | 0 | 25 | 100 |
| Strong | RF-1 | 15 ± 0.6 | 31 ± 1 | 49 ± 2.4 | 246 ± 16.7 | 276 ± 34 | 276 ± 21.6 | 7 ± 0.5 | 10 ± 0.8 | 10 ± 1.6 | 414 ± 47.4 | 532 ± 34.7 | 402 ± 40.5 |
|  | RF-102 | 19 ± 0.6 | 29 ± 1.7 | 50 ± 1.2 | 228 ± 10.5 | 226 ± 26.2 | 281 ± 20.3 | 6 ± 0.7 | 11 ± 1.9 | 10 ± 0.7 | 478 ± 96.6 | 478 ± 53.2 | 438 ± 19.8 |
|  | RF-15 | 20 ± 0.7 | 32 ± 1 | 52 ± 1.7 | 242 ± 11.6 | 341 ± 26.6 | 384 ± 19.6 | 8 ± 0.5 | 9 ± 0.7 | 11 ± 1.1 | 431 ± 67.8 | 517 ± 31.1 | 461 ± 36.5 |
|  | RF-16 | 20 ± 0.7 | 35 ± 1.2 | 59 ± 1.3 | 300 ± 20.3 | 381 ± 42 | 560 ± 44.3 | 6 ± 0.6 | 8 ± 0.5 | 9 ± 1.1 | 735 ± 79.9 | 604 ± 29.8 | 578 ± 87.2 |
|  | RF-2 | 17 ± 0.6 | 34 ± 1 | 60 ± 2.2 | 277 ± 21.4 | 372 ± 30.5 | 404 ± 15.3 | 9 ± 1.2 | 9 ± 1 | 12 ± 0.6 | 519 ± 67 | 516 ± 53.4 | 470 ± 59.2 |
|  | RF-23 | 19 ± 0.4 | 36 ± 1.5 | 54 ± 1.1 | 316 ± 19 | 443 ± 30.1 | 527 ± 33.8 | 3 ± 0.3 | 5 ± 0.4 | 4 ± 0.4 | 547 ± 42.8 | 502 ± 48.9 | 426 ± 51 |
|  | RF-29 | 20 ± 0.8 | 38 ± 0.7 | 54 ± 2.2 | 283 ± 11.1 | 424 ± 23.7 | 412 ± 21.8 | 8 ± 0.5 | 9 ± 1.1 | 9 ± 0.6 | 558 ± 70.1 | 500 ± 62.1 | 384 ± 40 |
|  | RF-3 | 21 ± 0.6 | 37 ± 1.2 | 51 ± 5 | 330 ± 14.4 | 382 ± 41.1 | 500 ± 54.3 | 6 ± 0.4 | 7 ± 0.8 | 9 ± 0.6 | 465 ± 66.8 | 462 ± 31.3 | 371 ± 80.5 |
|  | RF-7 | 14 ± 0.5 | 28 ± 1.2 | 45 ± 1.3 | 233 ± 16.3 | 264 ± 25.9 | 282 ± 15.8 | 7 ± 0.4 | 9 ± 2 | 7 ± 0.9 | 451 ± 66.5 | 469 ± 47.6 | 395 ± 21.3 |
|  | RF-94 | 18 ± 0.5 | 32 ± 1.4 | 53 ± 1.7 | 324 ± 15.2 | 343 ± 16.5 | 425 ± 35.1 | 7 ± 0.4 | 9 ± 1 | 11 ± 1.2 | 571 ± 44.4 | 462 ± 58.7 | 346 ± 40.3 |
|  | RZ-CAP | 18 ± 0.5 | 34 ± 1.8 | 56 ± 1.3 | 250 ± 16.2 | 297 ± 32.6 | 354 ± 12.9 | 6 ± 0.2 | 11 ± 2.4 | 10 ± 0.4 | 643 ± 73.3 | 461 ± 55.7 | 418 ± 43.5 |
|  |  |  |  |  |  |  |  |  |  |  |  |  |  |
| Moderate | BJ-HB1 | 17 ± 0.4 | 34 ± 1.1 | 52 ± 1.5 | 261 ± 14.3 | 343 ± 27.6 | 443 ± 38.9 | 3 ± 0.2 | 6 ± 0.8 | 7 ± 0.4 | 532 ± 44.9 | 554 ± 36.5 | 428 ± 69.5 |
|  | RF-11 | 16 ± 1.2 | 32 ± 1.4 | 54 ± 1.2 | 260 ± 21 | 348 ± 37 | 284 ± 43.5 | 7 ± 1 | 7 ± 0.8 | 13 ± 1 | 425 ± 41.2 | 463 ± 53.8 | 435 ± 47 |
|  | RF-20 | 20 ± 0.5 | 38 ± 1 | 49 ± 1 | 295 ± 17.3 | 339 ± 45.5 | 322 ± 11.8 | 4 ± 0.3 | 9 ± 2.2 | 8 ± 0.6 | 344 ± 52.8 | 477 ± 40.4 | 416 ± 36.6 |
|  | RF-22 | 20 ± 0.6 | 33 ± 1.2 | 58 ± 1.4 | 141 ± 45 | 405 ± 33.4 | 371 ± 34.7 | 5 ± 0.5 | 8 ± 3 | 10 ± 0.7 | 387 ± 75.4 | 474 ± 49.6 | 298 ± 74.7 |
|  | RF-226 | 15 ± 0.5 | 26 ± 2.2 | 43 ± 0.6 | 210 ± 10.9 | 176 ± 35.6 | 204 ± 19.3 | 8 ± 0.3 | 9 ± 1.5 | 12 ± 0.9 | 451 ± 98.1 | 588 ± 70.7 | 261 ± 32.8 |
|  | RF-27 | 16 ± 0.7 | 23 ± 1.3 | 50 ± 2 | 332 ± 13.2 | 323 ± 34.1 | 502 ± 47.9 | 5 ± 0.3 | 5 ± 0.4 | 5 ± 0.5 | 531 ± 44.5 | 476 ± 53.3 | 371 ± 43.9 |
|  | RF-34 | 19 ± 0.8 | 28 ± 2.8 | 43 ± 0.9 | 242 ± 31 | 231 ± 46.3 | 254 ± 12.5 | 7 ± 0.4 | 7 ± 0.8 | 7 ± 1 | 335 ± 40.1 | 435 ± 45.5 | 357 ± 21.3 |
|  | RF-40 | 17 ± 0.6 | 30 ± 1.4 | 54 ± 2.3 | 267 ± 29.3 | 282 ± 30.9 | 329 ± 16.4 | 5 ± 0.8 | 7 ± 0.7 | 10 ± 0.7 | 527 ± 46 | 482 ± 76.2 | 352 ± 50.5 |
|  | RF-43 | 15 ± 0.4 | 30 ± 2 | 47 ± 1.4 | 261 ± 26.2 | 296 ± 44 | 325 ± 20.4 | 6 ± 0.3 | 7 ± 0.8 | 7 ± 0.7 | 467 ± 71.2 | 575 ± 48.1 | 427 ± 57.8 |
|  | RF-89 | 18 ± 0.5 | 32 ± 1.1 | 51 ± 1.6 | 238 ± 18.2 | 271 ± 23.9 | 272 ± 19.4 | 7 ± 0.7 | 8 ± 1.2 | 8 ± 1 | 432 ± 49.9 | 423 ± 41.4 | 438 ± 44.5 |
|  | RF-97 | 19 ± 0.7 | 31 ± 0.8 | 50 ± 2.1 | 328 ± 16 | 335 ± 34.8 | 378 ± 26.9 | 4 ± 0.3 | 6 ± 0.7 | 7 ± 0.5 | 537 ± 95.3 | 532 ± 53.8 | 408 ± 57.6 |
|  |  |  |  |  |  |  |  |  |  |  |  |  |  |
| Weak | BJ-HB2 | 18 ± 0.6 | 30 ± 1.3 | 39 ± 1.6 | 228 ± 9.2 | 251 ± 26 | 239 ± 23.5 | 5 ± 0.5 | 5 ± 0.6 | 7 ± 0.6 | 613 ± 84.1 | 661 ± 55 | 421 ± 57.6 |
|  | N-9008 | 18 ± 0.5 | 34 ± 1.4 | 55 ± 1.4 | 267 ± 17.5 | 348 ± 31.5 | 366 ± 27.2 | 5 ± 0.1 | 7 ± 0.9 | 10 ± 2.2 | 532 ± 60.2 | 538 ± 18.2 | 398 ± 46.9 |
|  | N-9098 | 19 ± 0.6 | 30 ± 2.2 | 50 ± 0.9 | 212 ± 8.5 | 232 ± 32.4 | 229 ± 10.2 | 5 ± 0.4 | 9 ± 2.5 | 9 ± 1.3 | 379 ± 42.3 | 493 ± 47.3 | 422 ± 50.3 |
|  | N-FM001 | 21 ± 0.8 | 33 ± 0.6 | 50 ± 1.3 | 187 ± 20.6 | 242 ± 21.4 | 189 ± 23.4 | 7 ± 0.4 | 7 ± 0.7 | 10 ± 1.3 | 366 ± 53.1 | 434 ± 49.9 | 371 ± 41.5 |
|  | RF-103 | 12 ± 0.3 | 18 ± 1.2 | 25 ± 2.9 | 236 ± 47.9 | 304 ± 44.7 | 173 ± 44.7 | 4 ± 0.2 | 9 ± 4 | 7 ± 0.7 | 382 ± 46.1 | 417 ± 77 | 345 ± 51.5 |
|  | RF-206 | 19 ± 0.4 | 35 ± 0.9 | 53 ± 1.8 | 336 ± 46.6 | 407 ± 39.7 | 406 ± 32.4 | 5 ± 0.4 | 9 ± 1.8 | 8 ± 0.5 | 580 ± 68.2 | 615 ± 81.5 | 446 ± 72 |
|  | RF-229 | 16 ± 0.7 | 22 ± 0.9 | 35 ± 1.9 | 300 ± 7.9 | 270 ± 24.6 | 318 ± 19 | 4 ± 0.1 | 5 ± 0.6 | 6 ± 0.4 | 458 ± 41.6 | 452 ± 78.7 | 390 ± 34.8 |
|  | RF-4 | 19 ± 0.3 | 34 ± 1 | 51 ± 2.2 | 318 ± 6.2 | 426 ± 29.8 | 302 ± 25.1 | 6 ± 0.3 | 6 ± 0.7 | 7 ± 0.4 | 573 ± 49.9 | 487 ± 40.9 | 493 ± 54.6 |
|  | RF-91 | 21 ± 0.5 | 32 ± 2.1 | 48 ± 3.1 | 271 ± 9.1 | 249 ± 23.9 | 143 ± 26 | 5 ± 0.5 | 7 ± 0.8 | 9 ± 0.7 | 488 ± 42.9 | 367 ± 34.9 | 316 ± 42.4 |
|  | RF-93 | 20 ± 0.5 | 30 ± 1.3 | 48 ± 1.4 | 302 ± 21.3 | 302 ± 33.3 | 326 ± 29.9 | 5 ± 0.3 | 7 ± 1.4 | 7 ± 0.9 | 482 ± 54.3 | 433 ± 51.2 | 387 ± 32.9 |
|  | RZ-CAL | 19 ± 0.6 | 32 ± 1 | 51 ± 1.6 | 274 ± 12.8 | 314 ± 20.5 | 306 ± 33.8 | 6 ± 0.3 | 7 ± 0.5 | 9 ± 0.5 | 490 ± 67.7 | 528 ± 39.5 | 377 ± 60.5 |

Data are mean ± s.e.m (n=8 for 0 and 100 μmol m^−2^ s^−1^ of far red and n=16 for 25 μmol m^−2^ s^−1^ of far red)

*Table S1 Plant height, plant dry mass, leaf dry mass, stem dry mass, root dry mass, leaf area, shoot: root ratio, stomatal conductance and chlorophyll index of all genotypes grown with 0, 25 or 100 μmol m^−2^ s^−1^ of far red measured after 21 days of growth. (continued).*

| Group | Genotype | Plant dry mass | | | Leaf dry mass | | | Stem dry mass | | | Root dry mass | | | Shoot: root ratio | | |
| --- | --- | --- | --- | --- | --- | --- | --- | --- | --- | --- | --- | --- | --- | --- | --- | --- |
|  |  | (g) | | | (g) | | | (g) | | | (g) | | | (g g-1) | | |
|  | Far red | 0 | 25 | 100 | 0 | 25 | 100 | 0 | 25 | 100 | 0 | 25 | 100 | 0 | 25 | 100 |
| Strong | RF-1 | 0.7 ± 0.07 | 0.9 ± 0.08 | 1 ± 0.1 | 0.4 ± 0.04 | 0.5 ± 0.05 | 0.4 ± 0.05 | 0.1 ± 0.01 | 0.3 ± 0.02 | 0.4 ± 0.03 | 0.08 ± 0.01 | 0.08 ± 0.01 | 0.09 ± 0.01 | 7 ± 0.5 | 10 ± 0.8 | 10 ± 1.6 |
|  | RF-102 | 0.5 ± 0.05 | 0.6 ± 0.05 | 0.8 ± 0.06 | 0.3 ± 0.03 | 0.3 ± 0.03 | 0.3 ± 0.03 | 0.1 ± 0.01 | 0.1 ± 0.01 | 0.3 ± 0.02 | 0.08 ± 0.01 | 0.06 ± 0.01 | 0.07 ± 0 | 6 ± 0.7 | 11 ± 1.9 | 10 ± 0.7 |
|  | RF-15 | 0.9 ± 0.05 | 1.1 ± 0.1 | 1.4 ± 0.11 | 0.6 ± 0.03 | 0.6 ± 0.06 | 0.7 ± 0.06 | 0.2 ± 0.01 | 0.3 ± 0.02 | 0.6 ± 0.05 | 0.1 ± 0.01 | 0.12 ± 0.01 | 0.12 ± 0.01 | 8 ± 0.5 | 9 ± 0.7 | 11 ± 1.1 |
|  | RF-16 | 0.9 ± 0.07 | 1.2 ± 0.13 | 2.2 ± 0.23 | 0.5 ± 0.04 | 0.7 ± 0.09 | 1.1 ± 0.16 | 0.2 ± 0.01 | 0.3 ± 0.03 | 0.8 ± 0.07 | 0.14 ± 0.01 | 0.14 ± 0.02 | 0.21 ± 0.01 | 6 ± 0.6 | 8 ± 0.5 | 9 ± 1.1 |
|  | RF-2 | 0.9 ± 0.07 | 1.2 ± 0.11 | 1.6 ± 0.12 | 0.6 ± 0.06 | 0.7 ± 0.07 | 0.8 ± 0.06 | 0.1 ± 0.01 | 0.4 ± 0.03 | 0.7 ± 0.04 | 0.09 ± 0.01 | 0.12 ± 0.01 | 0.12 ± 0 | 9 ± 1.2 | 9 ± 1 | 12 ± 0.6 |
|  | RF-23 | 1.1 ± 0.06 | 1.3 ± 0.1 | 1.9 ± 0.11 | 0.6 ± 0.03 | 0.7 ± 0.05 | 0.8 ± 0.05 | 0.2 ± 0.01 | 0.4 ± 0.03 | 0.7 ± 0.04 | 0.28 ± 0.03 | 0.22 ± 0.03 | 0.39 ± 0.03 | 3 ± 0.3 | 5 ± 0.4 | 4 ± 0.4 |
|  | RF-29 | 0.9 ± 0.04 | 1.4 ± 0.08 | 1.4 ± 0.12 | 0.6 ± 0.02 | 0.8 ± 0.05 | 0.7 ± 0.06 | 0.2 ± 0.01 | 0.4 ± 0.02 | 0.6 ± 0.04 | 0.1 ± 0.01 | 0.17 ± 0.03 | 0.14 ± 0.01 | 8 ± 0.5 | 9 ± 1.1 | 9 ± 0.6 |
|  | RF-3 | 0.9 ± 0.06 | 1.2 ± 0.1 | 1.6 ± 0.43 | 0.6 ± 0.04 | 0.7 ± 0.06 | 0.8 ± 0.23 | 0.2 ± 0.01 | 0.4 ± 0.03 | 0.6 ± 0.14 | 0.12 ± 0.01 | 0.15 ± 0.02 | 0.17 ± 0.05 | 6 ± 0.4 | 7 ± 0.8 | 9 ± 0.6 |
|  | RF-7 | 0.5 ± 0.07 | 0.7 ± 0.11 | 0.8 ± 0.03 | 0.4 ± 0.04 | 0.4 ± 0.06 | 0.3 ± 0.02 | 0.1 ± 0.01 | 0.2 ± 0.03 | 0.3 ± 0.01 | 0.07 ± 0.01 | 0.1 ± 0.03 | 0.1 ± 0.01 | 7 ± 0.4 | 9 ± 2 | 7 ± 0.9 |
|  | RF-94 | 1 ± 0.07 | 1.2 ± 0.07 | 1.5 ± 0.15 | 0.6 ± 0.05 | 0.6 ± 0.05 | 0.7 ± 0.08 | 0.2 ± 0.01 | 0.3 ± 0.01 | 0.6 ± 0.05 | 0.12 ± 0.01 | 0.12 ± 0.01 | 0.13 ± 0.01 | 7 ± 0.4 | 9 ± 1 | 11 ± 1.2 |
|  | RZ-CAP | 1 ± 0.09 | 1.2 ± 0.17 | 1.7 ± 0.1 | 0.6 ± 0.06 | 0.6 ± 0.09 | 0.8 ± 0.05 | 0.2 ± 0.01 | 0.3 ± 0.05 | 0.7 ± 0.03 | 0.15 ± 0.01 | 0.12 ± 0.02 | 0.15 ± 0.01 | 6 ± 0.2 | 11 ± 2.4 | 10 ± 0.4 |
|  |  |  |  |  |  |  |  |  |  |  |  |  |  |  |  |  |
| Moderate | BJ-HB1 | 0.9 ± 0.08 | 1.2 ± 0.1 | 1.4 ± 0.16 | 0.5 ± 0.04 | 0.7 ± 0.06 | 0.7 ± 0.08 | 0.1 ± 0.01 | 0.3 ± 0.02 | 0.5 ± 0.05 | 0.22 ± 0.03 | 0.18 ± 0.02 | 0.19 ± 0.02 | 3 ± 0.2 | 6 ± 0.8 | 7 ± 0.4 |
|  | RF-11 | 0.9 ± 0.11 | 1.3 ± 0.13 | 1.3 ± 0.11 | 0.6 ± 0.07 | 0.7 ± 0.08 | 0.6 ± 0.06 | 0.1 ± 0.01 | 0.3 ± 0.03 | 0.6 ± 0.04 | 0.12 ± 0.02 | 0.17 ± 0.02 | 0.09 ± 0 | 7 ± 1 | 7 ± 0.8 | 13 ± 1 |
|  | RF-20 | 1 ± 0.06 | 1.2 ± 0.16 | 1.3 ± 0.07 | 0.6 ± 0.04 | 0.6 ± 0.09 | 0.6 ± 0.05 | 0.2 ± 0.01 | 0.3 ± 0.04 | 0.5 ± 0.01 | 0.19 ± 0.01 | 0.15 ± 0.03 | 0.13 ± 0.01 | 4 ± 0.3 | 9 ± 2.2 | 8 ± 0.6 |
|  | RF-22 | 1.2 ± 0.1 | 1.3 ± 0.14 | 1.4 ± 0.13 | 0.7 ± 0.07 | 0.7 ± 0.07 | 0.6 ± 0.07 | 0.2 ± 0.02 | 0.3 ± 0.03 | 0.6 ± 0.05 | 0.19 ± 0.01 | 0.2 ± 0.04 | 0.13 ± 0.01 | 5 ± 0.5 | 8 ± 3 | 10 ± 0.7 |
|  | RF-226 | 0.5 ± 0.04 | 0.6 ± 0.09 | 0.6 ± 0.03 | 0.3 ± 0.02 | 0.3 ± 0.06 | 0.2 ± 0.01 | 0.1 ± 0 | 0.1 ± 0.03 | 0.2 ± 0.01 | 0.05 ± 0 | 0.06 ± 0.01 | 0.04 ± 0 | 8 ± 0.3 | 9 ± 1.5 | 12 ± 0.9 |
|  | RF-27 | 1 ± 0.06 | 0.9 ± 0.09 | 1.5 ± 0.08 | 0.6 ± 0.03 | 0.5 ± 0.06 | 0.7 ± 0.05 | 0.2 ± 0.01 | 0.2 ± 0.01 | 0.5 ± 0.03 | 0.18 ± 0.01 | 0.17 ± 0.02 | 0.29 ± 0.02 | 5 ± 0.3 | 5 ± 0.4 | 5 ± 0.5 |
|  | RF-34 | 0.9 ± 0.17 | 0.8 ± 0.17 | 0.9 ± 0.08 | 0.5 ± 0.07 | 0.4 ± 0.08 | 0.3 ± 0.02 | 0.2 ± 0.09 | 0.2 ± 0.05 | 0.3 ± 0.01 | 0.11 ± 0.02 | 0.1 ± 0.03 | 0.14 ± 0.04 | 7 ± 0.4 | 7 ± 0.8 | 7 ± 1 |
|  | RF-40 | 0.9 ± 0.09 | 0.9 ± 0.11 | 1.1 ± 0.07 | 0.5 ± 0.07 | 0.5 ± 0.07 | 0.5 ± 0.03 | 0.1 ± 0.02 | 0.2 ± 0.02 | 0.5 ± 0.02 | 0.15 ± 0.01 | 0.12 ± 0.02 | 0.1 ± 0.01 | 5 ± 0.8 | 7 ± 0.7 | 10 ± 0.7 |
|  | RF-43 | 0.7 ± 0.1 | 0.9 ± 0.13 | 1 ± 0.09 | 0.4 ± 0.07 | 0.5 ± 0.08 | 0.4 ± 0.04 | 0.1 ± 0.01 | 0.2 ± 0.04 | 0.4 ± 0.04 | 0.11 ± 0.01 | 0.11 ± 0.01 | 0.12 ± 0.01 | 6 ± 0.3 | 7 ± 0.8 | 7 ± 0.7 |
|  | RF-89 | 0.7 ± 0.06 | 1 ± 0.11 | 1.1 ± 0.08 | 0.4 ± 0.04 | 0.6 ± 0.06 | 0.5 ± 0.04 | 0.1 ± 0.01 | 0.3 ± 0.02 | 0.5 ± 0.03 | 0.1 ± 0.01 | 0.14 ± 0.02 | 0.13 ± 0.02 | 7 ± 0.7 | 8 ± 1.2 | 8 ± 1 |
|  | RF-97 | 1.2 ± 0.09 | 1.1 ± 0.12 | 1.4 ± 0.11 | 0.7 ± 0.05 | 0.6 ± 0.07 | 0.7 ± 0.06 | 0.2 ± 0.01 | 0.3 ± 0.03 | 0.5 ± 0.04 | 0.22 ± 0.02 | 0.15 ± 0.02 | 0.17 ± 0.02 | 4 ± 0.3 | 6 ± 0.7 | 7 ± 0.5 |
|  |  |  |  |  |  |  |  |  |  |  |  |  |  |  |  |  |
| Weak | BJ-HB2 | 0.8 ± 0.06 | 0.8 ± 0.1 | 0.7 ± 0.06 | 0.5 ± 0.04 | 0.4 ± 0.06 | 0.3 ± 0.03 | 0.1 ± 0 | 0.2 ± 0.02 | 0.2 ± 0.02 | 0.14 ± 0.01 | 0.13 ± 0.02 | 0.09 ± 0.01 | 5 ± 0.5 | 5 ± 0.6 | 7 ± 0.6 |
|  | N-9008 | 1.2 ± 0.08 | 1.5 ± 0.12 | 1.4 ± 0.12 | 0.8 ± 0.05 | 0.8 ± 0.08 | 0.6 ± 0.06 | 0.2 ± 0.01 | 0.4 ± 0.03 | 0.6 ± 0.04 | 0.18 ± 0.01 | 0.19 ± 0.03 | 0.14 ± 0.02 | 5 ± 0.1 | 7 ± 0.9 | 10 ± 2.2 |
|  | N-9098 | 1 ± 0.06 | 0.9 ± 0.16 | 0.9 ± 0.04 | 0.6 ± 0.04 | 0.5 ± 0.08 | 0.3 ± 0.02 | 0.2 ± 0.01 | 0.3 ± 0.05 | 0.4 ± 0.02 | 0.16 ± 0.01 | 0.14 ± 0.03 | 0.09 ± 0.01 | 5 ± 0.4 | 9 ± 2.5 | 9 ± 1.3 |
|  | N-FM001 | 0.8 ± 0.07 | 0.9 ± 0.08 | 0.7 ± 0.1 | 0.4 ± 0.04 | 0.5 ± 0.05 | 0.3 ± 0.04 | 0.2 ± 0.01 | 0.3 ± 0.02 | 0.3 ± 0.04 | 0.1 ± 0.01 | 0.11 ± 0.01 | 0.08 ± 0.01 | 7 ± 0.4 | 7 ± 0.7 | 10 ± 1.3 |
|  | RF-103 | 0.6 ± 0.12 | 0.6 ± 0.07 | 0.4 ± 0.08 | 0.4 ± 0.08 | 0.4 ± 0.05 | 0.2 ± 0.05 | 0 ± 0.01 | 0.1 ± 0.01 | 0.1 ± 0.02 | 0.11 ± 0.02 | 0.1 ± 0.02 | 0.05 ± 0.01 | 4 ± 0.2 | 9 ± 4 | 7 ± 0.7 |
|  | RF-206 | 1.4 ± 0.08 | 1.3 ± 0.13 | 1.3 ± 0.12 | 0.9 ± 0.05 | 0.7 ± 0.07 | 0.6 ± 0.06 | 0.3 ± 0.02 | 0.4 ± 0.03 | 0.5 ± 0.04 | 0.21 ± 0.01 | 0.16 ± 0.03 | 0.13 ± 0.01 | 5 ± 0.4 | 9 ± 1.8 | 8 ± 0.5 |
|  | RF-229 | 0.8 ± 0.02 | 0.7 ± 0.06 | 0.7 ± 0.05 | 0.5 ± 0.02 | 0.4 ± 0.04 | 0.3 ± 0.02 | 0.1 ± 0 | 0.1 ± 0.01 | 0.2 ± 0.02 | 0.14 ± 0 | 0.12 ± 0.01 | 0.1 ± 0 | 4 ± 0.1 | 5 ± 0.6 | 6 ± 0.4 |
|  | RF-4 | 1.1 ± 0.11 | 1.5 ± 0.09 | 1.2 ± 0.09 | 0.7 ± 0.08 | 0.8 ± 0.06 | 0.5 ± 0.05 | 0.2 ± 0.01 | 0.4 ± 0.01 | 0.5 ± 0.03 | 0.16 ± 0.01 | 0.22 ± 0.03 | 0.13 ± 0 | 6 ± 0.3 | 6 ± 0.7 | 7 ± 0.4 |
|  | RF-91 | 0.9 ± 0.02 | 0.8 ± 0.1 | 0.7 ± 0.08 | 0.5 ± 0.01 | 0.4 ± 0.04 | 0.3 ± 0.04 | 0.2 ± 0 | 0.2 ± 0.02 | 0.3 ± 0.03 | 0.15 ± 0.01 | 0.13 ± 0.05 | 0.06 ± 0.01 | 5 ± 0.5 | 7 ± 0.8 | 9 ± 0.7 |
|  | RF-93 | 1 ± 0.11 | 0.9 ± 0.12 | 1.1 ± 0.01 | 0.6 ± 0.07 | 0.5 ± 0.07 | 0.5 ± 0.01 | 0.2 ± 0.02 | 0.2 ± 0.03 | 0.4 ± 0.01 | 0.15 ± 0.02 | 0.12 ± 0.02 | 0.13 ± 0.01 | 5 ± 0.3 | 7 ± 1.4 | 7 ± 0.9 |
|  | RZ-CAL | 1.1 ± 0.05 | 1.2 ± 0.11 | 1.3 ± 0.08 | 0.7 ± 0.03 | 0.7 ± 0.07 | 0.6 ± 0.05 | 0.2 ± 0.01 | 0.3 ± 0.02 | 0.5 ± 0.02 | 0.15 ± 0.01 | 0.14 ± 0.01 | 0.12 ± 0 | 6 ± 0.3 | 7 ± 0.5 | 9 ± 0.5 |
| Data are mean ± s.e.m (n=8 for 0 and 100 μmol m^−2^ s^−1^ of far red and n=16 for 25 μmol m^−2^ s^−1^ of far red) | | | | | | | | | | | | | | | | |

Table S2 Relative growth rate (RGR), net assimilation rate (NAR), leaf area ratio (LAR), specific leaf area (SLA) and leaf mass fraction (LMF) used in growth components analysis measured after 21 days of growth.

| Parameter | Light treatment |  | Group |  |
| --- | --- | --- | --- | --- |
|  |  | Strong | Moderate | Weak |
| RGR  (g g^-1^ d^-1^) | White + Red | 0.26 | 0.25 | 0.25 |
|  | White + Red + 25FR | 0.27 | 0.26 | 0.25 |
|  | White + Red + 100FR | 0.28 | 0.27 | 0.26 |
|  |  |  |  |  |
| NAR  (g cm^-2^ d^-1^) | White + Red | 8.3E-04 | 8.9E-04 | 9.8E-04 |
|  | White + Red + 25FR | 9.0E-04 | 9.0E-04 | 9.0E-04 |
|  | White + Red + 100FR | 1.0E-03 | 9.7E-04 | 9.2E-04 |
|  |  |  |  |  |
| LAR  (cm^2^ g^-1^) | White + Red | 326.7 | 301.9 | 275.7 |
|  | White + Red + 25FR | 310.7 | 296.3 | 305.5 |
|  | White + Red + 100FR | 281.9 | 282.6 | 298.7 |
|  |  |  |  |  |
| SLA  (cm^2^ g^-1^) | White + Red | 504.4 | 488.3 | 440.2 |
|  | White + Red + 25FR | 545.4 | 531.3 | 539.7 |
|  | White + Red + 100FR | 603.4 | 617.0 | 636.5 |
|  |  |  |  |  |
| LMF  (g g^-1^) | White + Red | 0.66 | 0.63 | 0.63 |
|  | White + Red + 25FR | 0.57 | 0.56 | 0.57 |
|  | White + Red + 100FR | 0.46 | 0.45 | 0.46 |


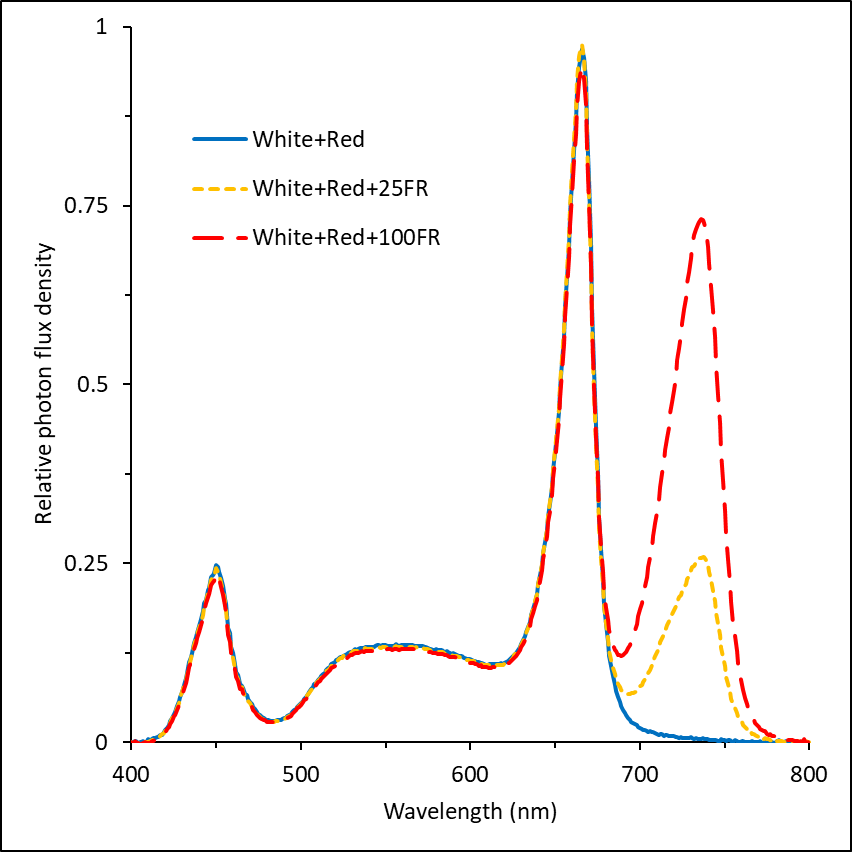


Figure S1 Spectral composition of light treatments provided by light-emitting diodes (LEDs) measured at the top of the canopy.
